# Risk Prediction Models of Cardiotoxicity in Patients With Breast Cancer: Multicenter Prospective CHECK HEART-BC Study

**DOI:** 10.1016/j.jacasi.2025.08.018

**Published:** 2025-12-19

**Authors:** Yosuke Terui, Kotaro Nochioka, Hideki Ota, Hiroshi Tada, Haruka Sato, Satoshi Miyata, Shigeru Toyoda, Shu Inami, Akihiro Nomura, Masaya Shimojima, Yasuhiro Izumiya, Yusuke Kodama, Takeshi Kitai, Kaoru Iwabuchi, Shu Suzuki, Daisuke Kitano, Keisuke Kida, Kiyotaka Shibuya, Takuya Oikawa, Takeru Nabeta, Toshiyuki Yano, Hiroyuki Iwano, Masayoshi Oikawa, Tatsuhiro Shibata, Yutaka Miura, Yoshito Ogihara, Nobuyuki Komiyama, Hiroshi Kato, Koji Higuchi, Sakiko Miyazaki, Yugo Yamashita, Satoshi Yasuda, Koichiro Sugimura

**Affiliations:** a Department of Cardiovascular Medicine, Tohoku University Graduate School of Public Health, Tokyo, Japan; b Department of Diagnostic Radiology, Tohoku University Graduate School of Public Health, Tokyo, Japan; c Department of Surgical Oncology, Tohoku University Graduate School of Public Health, Tokyo, Japan; d Teikyo University Graduate School of Public Health, Tokyo, Japan; e Department of Cardiovascular Medicine, Dokkyo Medical University, Mibu, Japan; f Department of Cardiovascular Medicine, Graduate School of Medical Sciences, Kanazawa University, Kanazawa, Ishikawa, Japan; g Department of Cardiovascular Medicine, Osaka Metropolitan University Graduate School of Medicine, Isaka, Japan; h Division of Cardiology, Department of Medicine, Showa University School of Medicine, Tokyo, Japan; i Department of Cardiovascular Medicine, Kobe City Medical Center General Hospital, Kobe, Japan; j Department of Heart Failure and Transplantation, National Cerebral and Cardiovascular Center, Osaka, Japan; k Cardiovascular Division, Osaki Citizen Hospital, Miyagi, Japan; l Department of Cardiology, KKR Tohoku Kosai Hospital, Sendai, Japan; m Division of Cardiology, Department of Medicine, Nihon University School of Medicine, Tokyo, Japan; n Department of Pharmacology, St Marianna University School of Medicine, Kawasaki, Japan; o Department of Cardiology, Saka General Hospital, Shiogama, Japan; p Department of Cardiovascular Medicine, Kesennuma City Hospital, Kesennuma, Japan; q Department of Cardiovascular Medicine, Kitasato University School of Medicine, Sagamihara, Kanagawa, Japan; r Department of Cardiovascular, Renal, and Metabolic Medicine, Sapporo Medical University School of Medicine, Sapporo, Japan; s Department of Cardiovascular Medicine, Faculty of Medicine and Graduate School Medicine, Hokkaido University, Sapporo, Japan; t Department of Cardiovascular Medicine, Fukushima Medical University, Fukushima, Japan; u Division of Cardiovascular Medicine, Department of Internal Medicine, Kurume University School of Medicine, Kurume, Japan; v Department of Cardiovascular Medicine, Tohoku Medical and Pharmaceutical University, Miyagi, Japan; w Department of Cardiology and Nephrology, Mie University Graduate School of Medicine, Tsu, Japan; x Department of Cardiovascular Medicine, St Luke’s International Hospital, Tokyo, Japan; y Division of Onco-Cardiology, Miyagi Cancer Center, Miyagi, Japan; z Department of Cardiovascular Medicine and Hypertension, Graduate School of Medicine and Dental Sciences, Kagoshima University, Kagoshima, Japan; aa Department of Cardiovascular Biology and Medicine, Juntendo University Graduate School of Medicine, Tokyo, Japan; bb Department of Cardiovascular Medicine, Graduate School of Medicine, Kyoto University, Kyoto, Japan; cc Department of Cardiology, International University of Health and Welfare Narita Hospital, Narita, Japan

**Keywords:** cardio-oncology, cardiomyopathy, chemotherapy, predictive model

## Abstract

**Background:**

Early detection and treatment of cardiotoxicity are essential for reducing cardiac events. However, a reliable predictive model for cardiotoxicity in patients with breast cancer receiving chemotherapy is lacking.

**Objectives:**

In this study, we aimed to develop a risk prediction model and establish effective surveillance for cardiotoxicity in patients with breast cancer undergoing chemotherapy.

**Methods:**

Patients with breast cancer scheduled for neoadjuvant and/or adjuvant chemotherapy were prospectively screened at 25 participating institutions between August 2017 and March 2020. Cardiotoxicity was defined as a reduction in left ventricular ejection fraction of >10% from baseline to a value <53%.

**Results:**

The study included 559 chemotherapy-naïve female patients. Cardiotoxicity was observed in 46 of 559 patients (8.2%) during a median follow-up period of 366 days (Q1-Q3: 365-367 days). The CHECK HEART (Comprehensive Heart Imaging to Evaluate Cardiac Damage Linked With Chemotherapy in Breast Cancer Patients) score consisted of 6 variables: heart rate, left ventricular global longitudinal strain, left ventricular end-systolic and end-diastolic diameters, right ventricular fractional area change, and treatment with anthracycline and trastuzumab. The time-dependent area under the receiver operating characteristic curve (AUC) at 12 months based on pretreatment data showed acceptable accuracy (AUC: 0.82; 95% CI: 0.76-0.89).

**Conclusions:**

The developed multivariable risk prediction models can accurately predict cardiotoxicity and support effective surveillance in patients with breast cancer receiving chemotherapy.

Breast cancer is the most frequently diagnosed cancer among women, and its incidence is increasing in most countries.^1^ Chemotherapy with anthracyclines and/or human epidermal growth factor receptor type 2 inhibitors, used to reduce the risk of recurrence, has improved the 5-year survival rate of patients with breast cancer by approximately 80% worldwide, resulting in a large population of long-term survivors.^2^, ^3^, ^4^ Taxanes are also effective against breast cancer when used with anthracyclines and human epidermal growth factor receptor type 2 inhibitors^5^; however, despite their high efficacy, they can cause cardiotoxicity, a major clinical concern.^6^, ^7^, ^8^ Early detection and treatment of cardiotoxicity play critical roles in reducing cardiac events, and several guidelines recommend systematic cardiac surveillance, including personalized cardiotoxicity risk assessment, biomarkers, and echocardiography before and during treatment.^9^, ^10^, ^11^, ^12^, ^13^ Although the utility of each surveillance method has been reported individually, a reliable predictive model for cardiotoxicity in patients with breast cancer receiving chemotherapy is lacking.^14^, ^15^, ^16^, ^17^

This multicenter prospective study CHECK HEART-BC (Comprehensive Heart Imaging to Evaluate Cardiac Damage Linked With Chemotherapy in Breast Cancer Patients) aimed to develop a robust prediction model and establish an effective surveillance of cardiotoxicity through systematic cardiac function evaluation.

## Methods

### Study design

**Figure 1:**
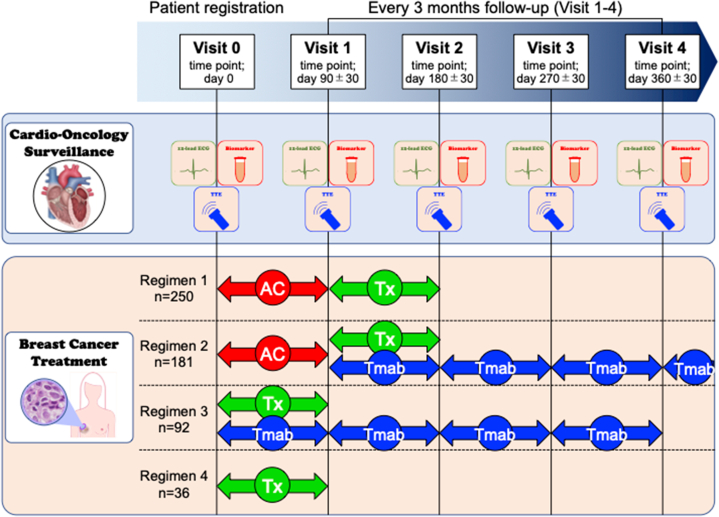
Study Protocol Cardio-oncology surveillance, including echocardiography, 12-lead electrocardiogram (ECG), and biomarkers, was performed every 3 months (visits 0 to 4). Breast cancer treatment was categorized into 4 regimens: anthracycline (AC) and taxane (Tx) (regimen 1; n = 250); anthracycline, trastuzumab (Tmab), and taxane (regimen 2; n = 181); trastuzumab and taxane (regimen 3; n = 92); and taxane alone (regimen 4; n = 36). Site visits were scheduled before chemotherapy (baseline, Visit 0), and every 3 months after chemotherapy initiation [Visits 1 (90 ± 30 days) to 4 (360 ± 30 days)]. Visit 0, baseline; Visits 1–4, visits at 3, 6, 9, and 12 months after chemotherapy initiation, respectively. TTE = transthoracic echocardiogram.

CHECK HEART-BC was a prospective, multicenter observational study aimed at identifying clinical features and developing a prediction model for cardiotoxicity in patients with breast cancer (the University Hospital Medical Information Network ID 000032401). Patients were consecutively enrolled at 25 participating institutions in Japan. Echocardiography, high-sensitivity cardiac troponin T or I (hs-TnT/I), B-type natriuretic peptide (BNP), N-terminal pro-BNP, and 12-lead electrocardiography were performed according to the follow-up protocol to evaluate cardiac function (Figure 1). The study protocol was approved by the respective institutional ethics committee (identifier number of representative institution 2017-1-866), and written informed consent was obtained from all patients. The study was conducted in accordance with the ethical principles of the Declaration of Helsinki.

### Patient enrollment and data collection

Patients with breast cancer scheduled for neoadjuvant and/or adjuvant chemotherapy between August 2017 and March 2020 were prospectively enrolled. Clinical data, including patient and tumor characteristics, laboratory data, and echocardiographic and electrocardiography parameters, were collected at baseline (visit 0) by clinical research coordinators or investigators at each institution using electronic data capture systems. Cardio-oncology surveillance, including echocardiography, 12-lead electrocardiography, and biomarkers, was performed every three months after chemotherapy initiation (visits 1 [90 ± 30 days] to 4 [360 ± 30 days]). Patients were excluded if they did not undergo echocardiography before chemotherapy, were not treated with chemotherapy, or had received prior chemotherapy for any cancer.

### Definition of cardiotoxicity

For early and accurate detection of subsequent cardiotoxicity, the risk prediction model was based on clinical parameters obtained before chemotherapy initiation. The primary outcome, cardiotoxicity development, was defined as a reduction of >10% point in left ventricular ejection fraction (LVEF) from baseline to a value <53% measured by echocardiography, in accordance with the European Society of Cardiology 2016 position paper on cancer treatments and cardiovascular toxicity.^8^^,^^18^ The secondary outcome was a composite of cardiac death, myocardial infarction, symptomatic heart failure, and arrhythmia requiring additional cardiac treatment. Cardioprotective treatment following the development of cardiotoxicity was administered at the discretion of the attending physician.

### Derivation of the risk prediction model

The risk prediction model was derived using patient characteristics and clinical data at baseline (visit 0). Supplemental Table 1 shows the relationship between the visit 0 variables and cardiotoxicity development using univariable Cox regression analysis, as well as the rate of missing values. The variables for the risk prediction model were selected according to following steps: 1) to avoid reducing the model discrimination and biasing the results, all the missing values were replaced using multiple imputation; 2) multivariable Cox regression analysis was performed with variables selection using backward elimination; 3) steps 1 and 2 were repeated 20 times, and the top 5 variables with the highest frequency of selection were chosen (Supplemental Table 2); and 4) beta coefficients from multivariable Cox regression analysis of each imputation dataset were combined using Rubin’s rule and risk score points were calculated. Additionally, radiotherapy and sequential therapy with anthracyclines and trastuzumab are known to be associated with a high risk of cardiotoxicity. The accuracy of the risk prediction model, including different combinations of the 5 selected variables and/or radiotherapy and/or sequential therapy, was evaluated. Finally, the most accurate risk prediction model, termed the CHECK HEART score, consisted of the 5 variables selected through repeated multivariable analysis plus cancer therapy variables identified through literature review.

For risk stratification, the participants were divided into 3 groups based on the model’s risk scores using cut-off values derived from the classification and regression trees (CART) analysis.^19^ The CART method, a decision tree methodology, identifies relationships between dependent variables and explanatory variables. In this analysis, risk score points were treated as explanatory variables to efficiently stratify populations regarding the development of cardiotoxicity.

To evaluate the accuracy and clinical implications of the risk prediction models, time-dependent area under the receiver operating characteristic curve (AUC) analyses were performed using both the raw risk score points and the categorical groups defined by the CART method-based cut-off values.^20^

### Statistical analysis

The normality of continuous variables was evaluated using normal Q-Q plots. Continuous variables are presented as mean ± SD or median with 25th-75th percentiles (Q1-Q3). Serum levels of hs-TnT/I and plasma levels of BNP and N-terminal pro-BNP were analyzed after logarithmic transformation to normalize their distributions. Post hoc pairwise comparisons of the temporal changes in each variable from visit 0 to visit 4 were performed using Dunnett's test (entire cohort and patients without cardiotoxicity) or Steel's test (patients with cardiotoxicity), with visit 0 as the reference. CART analysis was used to determine the cut-off values of risk score points for developing cardiotoxicity in the risk models.^18^ Cox proportional hazards models were applied to analyze the likelihood of developing cardiotoxicity, with results presented as HRs with 95% CIs. The proportional hazards assumption was tested using scaled Schoenfeld residuals. The minimum *P* values exceeded 0.05, indicating that the null hypotheses of proportional hazards for all covariates was not rejected. Time-dependent AUC curve analyses were performed to compare the accuracy of the risk prediction model using Uno’s concordance index. A competing risk analysis was performed using the Fine-Gray competing risk model to eliminate the effect of all-cause mortality on outcomes during follow-up. A cumulative incidence curve was generated using the Fine-Gray model to estimate the time to cardiotoxicity development adjusted competing risks. Internal statistical validation of the prediction model was assessed through a simulation study involving iterative random partitioning of data into training and validation sets as follows: 1) all 559 participants were randomly divided — 373 (66.7%) for training and 186 (33.3%) for validation sets, with the validation set entirely excluded during model training; 2) multivariable Cox regression analysis was applied to the training set to develop the original prediction model; 3) risk score points were calculated and the time-dependent AUC analyses were performed in both training and validation sets to evaluate model accuracy; and 4) steps 1-3 were repeated 1,000 times to validate the mean time-dependent AUC across the original, training, and validation sets.

Statistical significance was set at *P* < 0.05. All analysis was performed using R software, version 4.2.2 (R Foundation for Statistical Computing).

## Results

### Patient demographics and characteristics

**Figure 2:**
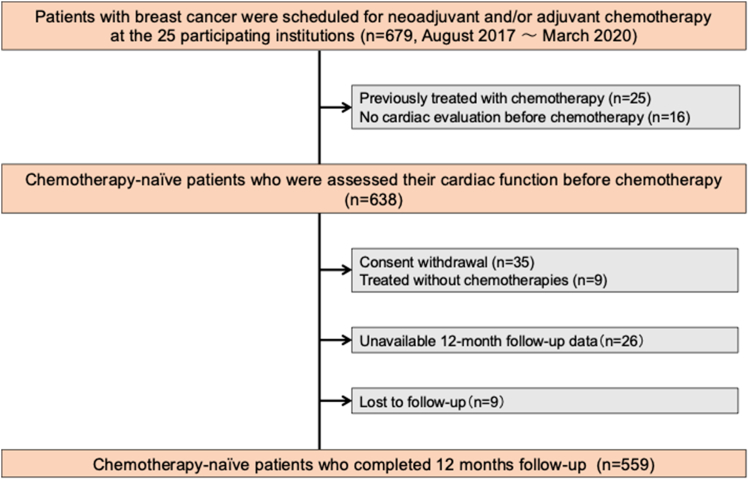
Patient Flowchart Of 679 prospectively enrolled patients with breast cancer scheduled for neoadjuvant and/or adjuvant chemotherapy, 41 were excluded based on predefined criteria. Ultimately, 559 chemotherapy-naïve patients were included.

Figure 2 shows the patient selection flowchart. Of 679 prospectively enrolled patients with breast cancer, 41 were excluded based on the exclusion criteria. An additional 79 patients were excluded due to consent withdrawal, unavailable 12-month follow-up data, or loss to follow-up. Ultimately, 559 chemotherapy-naïve patients were included in the analysis. Table 1 presents the baseline patient characteristics. The mean patient age was 56 ± 12 years, and all patients were female. The most prevalent comorbidity was dyslipidemia (n = 151 of 559, 27.0%), followed by hypertension (n = 140 of 559, 25.0%). Thirty-seven patients (n = 37 of 559, 6.6%) had a history of heart disease. Sixteen patients (n = 16 of 559, 2.9%) were receiving oral beta blockers and 80 patients (n = 80 of 559, 14.3%) were receiving renin-angiotensin system inhibitors due to comorbidities. A total of 431 patients (n = 431 of 559, 77.1%) were treated with anthracycline and 273 (n = 273 of 559, 48.8%) were treated with trastuzumab. Sequential therapy was administered to 181 patients (n = 181 of 559, 32.4%).

**Table 1:** Baseline Patient Characteristics

	Overall (N = 559)	Patients With Cardiotoxicity (n = 46)	Patients Not Having Cardiotoxicity at Their Last Follow-Up Visit (n = 513)
Demography			
Age, y	56 ± 12 (range: 28-86)	56 ± 12	56 ± 11
Female	559 (100)	46 (100)	513 (100)
Body mass index, kg/m^2^	23.3 ± 4.3	23.4 ± 4.3	22.9 ± 3.8
Cardiovascular risk factors			
Hypertension	140 (25.0)	9 (19.6)	131 (25.5)
Dyslipidemia	151 (27.0)	13 (28.3)	138 (28.3)
Diabetes mellitus	52 (9.3)	4 (8.7)	48 (9.4)
Current or ex-smoker	86 (15.4)	6 (13.0)	80 (15.6)
History of heart disease	37 (6.6)	5 (10.9)	32 (6.2)
Medication			
Beta-blocker	16 (2.9)	3 (6.5)	13 (2.5)
RAS inhibitors	80 (14.3)	6 (13.0)	74 (14.5)
Statins	96 (17.2)	9 (19.6)	87 (17.0)
Breast cancer characteristics			
Left side	270 (48.6)	24 (53.3)	246 (48.2)
Right side	271 (48.8)	21 (46.7)	250 (49.0)
Bilateral	14 (2.5)	0 (0.0)	14 (2.8)
Early stage	375 (67.1)	34 (73.9)	341 (66.5)
Triple negative cancer	117 (21.0)	5 (10.9)	112 (21.9)
Breast cancer therapy			
Surgery	513 (91.8)	43 (93.5)	470 (91.6)
Radiotherapy	318 (57.7)	33 (71.7)	285 (56.4)
Endocrine therapy	310 (55.8)	28 (60.9)	282 (55.4)
AC	431 (77.1)	39 (84.8)	392 (76.4)
AC dose, mg/m^2^	259 ± 40	264 ± 33	259 ± 41
Tmab	273 (48.8)	27 (58.7)	246 (48.0)
AC + Tmab	181 (32.4)	20 (43.5)	161 (31.4)
Others	36 (6.5)	0 (0.0)	36 (7.1)

Values are mean ± SD or n (%).

AC = anthracycline; RAS = renin-angiotensin-aldosterone system; Tmab = trastuzumab.

Cardiotoxicity was observed in 46 of 559 patients (8.2%; 85.9 per 1,000 person-years; 95% CI: 6.2-10.8) during a median follow-up period of 366 days (Q1-Q3: 365-367 days) (Supplemental Figure 1). Forty-four patients (n = 44 of 559, 95.7%) were asymptomatic, and only 2 were hospitalized due to symptomatic heart failure. Most patients who developed cardiotoxicity were treated with oral cardioprotective drugs, whereas patients with symptomatic heart failure were treated with diuretics at the discretion of the attending physician. There were 17 deaths from breast cancer and no deaths from other causes during the follow-up period. Table 1 compares the baseline characteristics of patients with and without cardiotoxicity. The subgroups were similar in age, body weight, and cardiovascular risk factors. Among the 46 patients with cardiotoxicity, 39 (n = 39 of 46, 84.8%) were treated with anthracycline, 27 (n = 27 of 46, 58.7%) with trastuzumab, and 20 (n = 20 of 46, 43.5%) with both agents. Supplemental Figure 2D shows a higher incidence of cardiotoxicity in patients receiving sequential therapy (anthracycline and trastuzumab) compared with those not receiving sequential therapy (log-rank test, *P* = 0.090), although the difference was not statistically significant. No patient developed cardiotoxicity after treatment with taxanes alone. Additionally, secondary outcomes included 4 arrhythmia events and 2 heart failure events during the follow-up period (Supplemental Table 3).

### Temporal changes in clinical data

Table 2 summarizes the temporal changes in the clinical data, including biomarker analysis, 12-lead electrocardiography, and echocardiography, for the entire cohort. Serum hs-Tn levels increased significantly, whereas plasma levels of BNP and N-terminal pro-BNP remained stable throughout the follow-up period. One hundred ninety-two of 199 patients (96.5%) with elevated serum hs-Tn levels 3 months after chemotherapy initiation (visit 1) had received anthracycline. A transient increase in heart rate was observed, which returned to baseline levels by visit 3. Slight end-diastolic and end-systolic left ventricular (LV) dilatation, and significant deterioration in LV and right ventricular (RV) systolic functions were noted at 360 days (visit 4) compared to baseline, including reduction in LVEF (from 67.6% ± 5.6% to 61.6% ± 6.3%; *P* < 0.001), left ventricular global longitudinal strain (LVGLS) (–21.3% ± 2.1% to –20.1% ± 2.5%; *P* < 0.001), and right ventricular fractional area change (RVFAC) (45.7% ± 6.5% to 44.3% ± 6.4%; *P* = 0.048). Mitral annular early diastolic velocity (e′) also declined (9.5 ± 2.9 cm/s to 9.0 ± 2.5 cm/s; *P* < 0.001), whereas peak E-wave velocity/e’ remained unchanged (7.8 ± 2.5 to 7.9 ± 2.5; *P* = 0.75). Similar trends were observed in patients without cardiotoxicity (Supplemental Table 4), whereas more pronounced temporal changes were noted in patients with cardiotoxicity (Supplemental Table 5).

**Table 2:** Temporal Changes in Clinical Data During Follow-Up^a^

	Visit 0 (Day 0) (n = 559)	Visit 1 (Day 90 ± 30) (n = 554)	Visit 2 (Day 180 ± 30) (n = 550)	Visit 3 (Day 270 ± 30) (n = 543)	Visit 4 (Day 360 ± 30) (n = 542)
Biomarker					
hs-TnT, ng/mL	0.005 (0.003-0.006)	0.013 (0.007-0.022)^b^	0.009 (0.006-0.013)^b^	0.007 (0.005-0.01)^b^	0.006 (0.005-0.008)^b^
hs-TnI, ng/mL	0.01 (0.004-0.01)	0.01 (0.01-0.017)^b^	0.01 (0.01-0.012)^c^	0.01 (0.008-0.01)	0.01 (0.009-0.01)
BNP, pg/mL	15.2 (8.2-24.9)	12.3 (6.3-24.7)	12.4 (6.9-21.9)	13.5 (8.0-23.3)	12.3 (7.0-21.5)
NT-proBNP, pg/mL	75.0 (34.1-100)	63.1 (30.1-107)	58.7 (33.7-114)	61.0 (37.0-105)	60.0 (31.1-106)
12-lead ECG					
Heart rate, beats/min	71.4 ± 11.1	78.5 ± 12.4^b^	76.0 ± 11.8^b^	71.0 ± 10.6	71.0 ± 10.9
PR interval, ms	156 ± 22	155 ± 21	158 ± 22	160 ± 23^c^	160 ± 23^b^
QRS interval, ms	92 ± 12	91 ± 11	91 ± 11	92 ± 11	92 ± 12
Corrected QT interval, ms	419 ± 22	426 ± 25^b^	427 ± 24^b^	425 ± 22^b^	424 ± 23^b^
Echocardiography					
LVDd, mm	43.5 ± 4.1	44.3 ± 4.2^b^	44.4 ± 4.3^b^	44.4 ± 4.1^b^	44.3 ± 4.3^c^
LVDs, mm	27.5 ± 3.4	28.5 ± 3.5^b^	28.9 ± 3.7^b^	28.8 ± 3.7^b^	28.8 ± 4.1^b^
LVEDVi, mL/m^2^	83.5 ± 14.9	86.8 ± 16.2^b^	86.9 ± 16.4^b^	87.1 ± 15.7^b^	86.6 ± 16.6^b^
LVESVi, mL/m^2^	32.7 ± 7.9	35.2 ± 9.1^b^	36.2 ± 9.9^b^	36.0 ± 10.0^b^	36.2 ± 10.6^b^
LVEF, %	67.6 ± 5.6	63.3 ± 5.2^b^	62.4 ± 5.7^b^	62.1 ± 5.9^b^	61.6 ± 6.3^b^
LAVI, mL/m^2^	25.2 ± 8.3	27.5 ± 8.7^b^	27.1 ± 8.5^b^	26.9 ± 8.7^b^	26.6 ± 8.7^c^
E/A	1.11 ± 0.4	1.09 ± 0.4	1.08 ± 0.4	1.08 ± 0.4	1.04 ± 0.4
e’, cm/s	9.5 ± 2.9	9.6 ± 2.6	9.4 ± 2.5	9.3 ± 2.5	9.0 ± 2.5^b^
E/e’	7.8 ± 2.5	7.8 ± 2.3	7.8 ± 2.4	7.7 ± 2.5	7.9 ± 2.5
LVGLS, %	−21.3 ± 2.1	−20.2 ± 2.5^b^	−20.2 ± 2.5^b^	−20.1 ± 2.5^b^	−20.1 ± 2.5^b^
RVFAC, %	45.7 ± 6.5	45.1 ± 6.2	45.0 ± 6.3	44.9 ± 6.5	44.3 ± 6.4^b^
TAPSE, mm	21.6 ± 3.9	21.6 ± 3.6	21.6 ± 3.8	21.4 ± 3.8	21.0 ± 3.6^c^

Values are mean ± SD or median (Q1-Q3).

BNP = B-type natriuretic peptide; ECG = electrocardiogram; hs-TnI = high-sensitive cardiac troponin I; hs-TnT = high-sensitive cardiac troponin T; LAVI = left atrial volume index; LVDd = left ventricular diameters in diastole; LVDs = left ventricular diameters in systole; LVEDVi = left ventricular end-diastolic volume index; LVEF = left ventricular ejection fraction; LVESVi = left ventricular end-systolic volume index; LVGLS = left ventricular global longitudinal strain; NT-proBNP = N-terminal pro−B-type natriuretic peptide; RVFAC = right ventricular fractional area change; TAPSE = tricuspid annular plane systolic excursion.

a The temporal changes in each data from visit 0 to visit 4 were compared by Dunnett’s method using the data at visit 0 as a reference.

b *P*<0.01.

c *P*<0.05.

### Risk prediction models based on the CHECK HEART score

**Figure 3:**
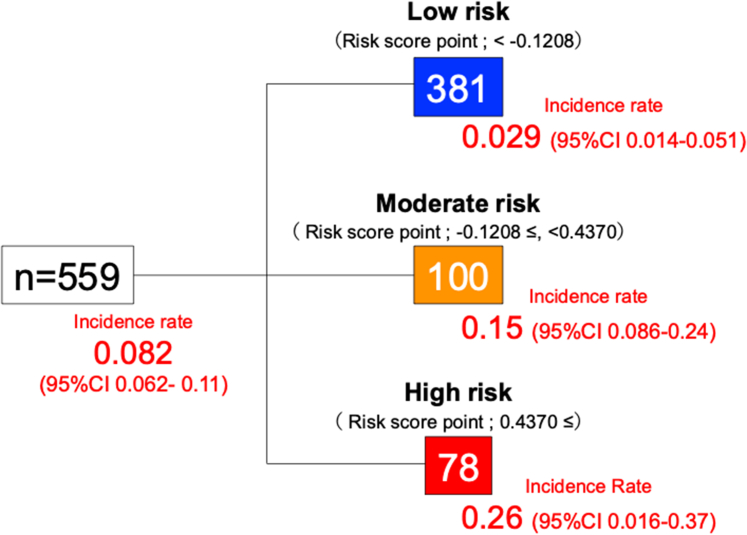
Risk Stratification Based on the CHECK HEART Score Participants (n = 559) in risk prediction model were divided into 3 risk-level groups based on their risk scores and cut-off values derived from classification and regression tree analysis. The incidence rates in the high-risk groups were notably higher than in the other groups.

**Figure 4:**
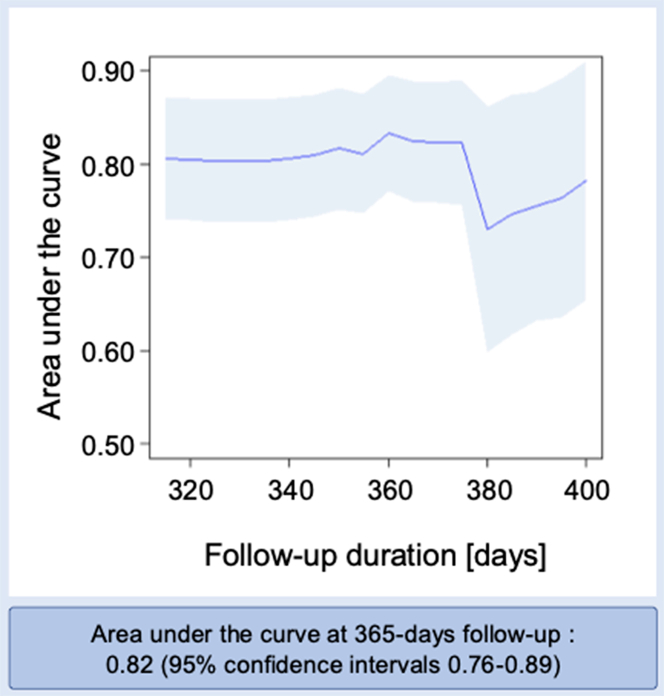
The CHECK HEART Score: Time-Dependent Area Under the Curve The CHECK HEART score was derived using patient characteristics and clinical data at baseline (visit 0). The blue line and zone show the time-dependent area under the receiver operating characteristic curve (time-dependent AUC) and 95% CI. The time-dependent AUC at 12 months was 0.82 (95%CI 0.76-0.89).

Supplemental Table 2 shows the frequency of variables selected in 20 repeated multivariable Cox regression analyses, with the top 5 variables used to develop the risk prediction model. Table 3 presents the time-dependent AUCs of models incorporating different combinations of the 5 variables selected based on multivariable analysis and/or radiotherapy and/or sequential therapy selected based on a literature review was evaluated. The most accurate model included the 5 selected variables plus sequential therapy with anthracycline and trastuzumab (Table 3). Table 4 shows multivariable Cox regression models, and the risk score was subsequently calculated based on beta coefficients. Furthermore, participants were stratified into low-, moderate-, and high-risk groups according to model risk scores using CART-derived cut-off values (Figure 3). The incidence of cardiotoxicity exceeded 25% in high-risk group. A summary of these models is provided in Table 5. In the high-risk group, the incidence rates of cardiotoxicity were notably higher than those in the low-risk group. Both the moderate- and high-risk groups had significantly higher HRs of developing cardiotoxicity, using the low-risk group as the reference. As shown in Figure 4, the time-dependent AUC of the raw risk score points was 0.80 or greater over the first 12 months, and the performance of the categories defined by CART methods was comparable. The final risk prediction model consisted of 6 variables: heart rate, LV end-systolic and end-diastolic diameters, LVGLS, RVFAC, and treatment with anthracycline and trastuzumab. The formula for the CHECK HEART score was as follows:

**Table 3:** The Time-Dependent AUCs of Different Combinations of the 5 Selected Variables and/or Cancer Therapy

Variable Combinations	Follow-up Duration From Chemotherapy Initiation, days				
	320	340	365	380	400
5 selected variables (heart rate, LVDd, LVDs, LVGLS, and RVFAC)	0.79	0.79	0.81	0.71	0.74
5 selected variables + sequential therapy + radiotherapy	0.81	0.81	0.81	0.71	0.76
5 selected variables + sequential therapy	0.80	0.80	0.82	0.72	0.78
5 selected variables + radiotherapy	0.80	0.80	0.81	0.70	0.73

AUC = area under the curve; other abbreviations as in Table 2.

**Table 4:** Multivariable Cox Regression Models for the Development of Cardiotoxicity

	Multivariable Analysis		
	HR (95% CI)	β Coefficient (Standard Error)	*P* Value
Echocardiography (at baseline, visit 0)			
LVDd, mm	0.96 (0.86-1.08)	−0.0384 (0.059)	0.52
LVDs, mm	1.23 (1.07-1.41)	0.209 (0.071)	0.005
LVGLS, %	1.22 (1.04-1.43)	0.199 (0.081)	0.020
RVFAC, %	0.95 (0.90-1.00)	−0.0540 (0.028)	0.059
12-lead ECG (at baseline, visit 0)			
Heart rate, beats/min	1.03 (1.00-1.05)	0.0269 (0.012)	0.034
Breast cancer treatment			
Anthracycline with trastuzumab	1.81 (0.99-3.29)	0.592 (0.31)	0.060

Multivariable predictors in the derivation cohort and the formula for calculating the CHECK HEART score. The CHECK HEART score = (0.0269 × [heart rate at baseline, beats/min]) – (0.0384 × [LVDd at baseline, mm]) + (0.209 × [LVDs at baseline, mm]) + (0.199 × [LVGLS at baseline, %]) – (0.0540 × [RVFAC at baseline, %]) + (0.592 × treated with anthracycline and trastuzumab).

Abbreviations as in Table 2.

**Table 5:** Summary of the CHECK HEART Score

Group	The Incidence of Cardiotoxicity Event/Sample Size (%)	HR (95% CI)	*P* Value
	46/559 (8.2)		
Low risk	11/381 (2.9)	Reference	NA
Moderate risk	15/100 (15.0)	5.89 (2.70-12.9)	<0.001
High-risk	20/78 (25.6)	11.5 (5.43-24.2)	<0.001

NA = not applicable.

CHECK HEART score = (0.0269 × [heart rate at baseline, beats/min]) − (0.0384 × [left ventricular diameters in diastole at baseline, mm]) + (0.209 × [left ventricular diameters in systole at baseline, mm]) + (0.199 × [LVGLS at baseline, %]) − (0.0540 × [RVFAC at baseline, %]) + (0.592 × treated with anthracycline and trastuzumab).

**Figure 5:**
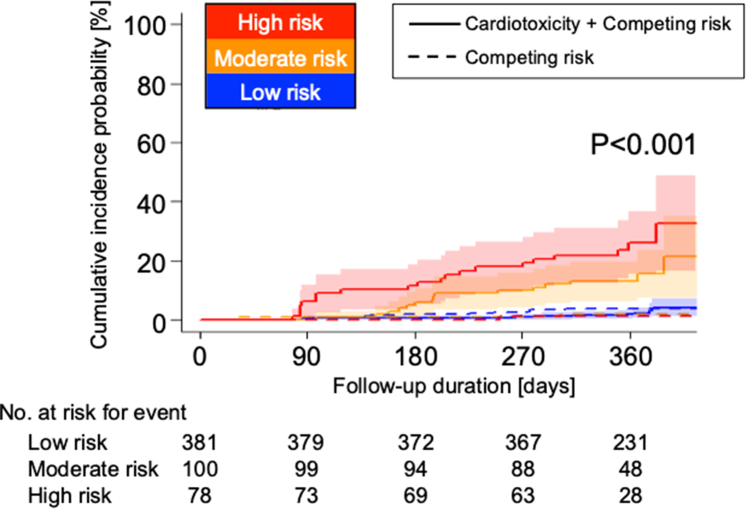
Cumulative Incidence Curve of Cardiotoxicity Using the CHECK HEART Scores Cumulative incidence curves using the Fine-Gray model showing the incidence of cardiotoxicity in each risk group based on the CHECK HEART score. The solid lines show the incidence of cardiotoxicity and a competing risk (all-cause death), and the dashed lines show the incidence of a competing risk (all-cause death).

The Kaplan–Meier curves of the risk prediction model categorized by CART methods showed a significant difference in the incidence of cardiotoxicity (Figure 5).

Several validation studies were conducted to evaluate the usefulness of the CHECK HEART score. First, the internal validation showed that the mean time-dependent AUCs at 12 months was 0.70 ± 0.01 both in training and validation sets. Second, the accuracy of the CHECK HEART score was demonstrated by evaluating various definitions of cardiotoxicity (Supplemental Figure 3).^9^^,^^11^^,^^15^^,^^21^ Using the new definition of cancer therapy–related cardiac dysfunction from the latest European Society of Cardiology guidelines on cardio-oncology, cardiotoxicity occurred in 28 of 559 patients (5.0%), with consistent time-dependent AUC values (0.83; 95% CI: 0.77-0.90). Furthermore, a competing risk model incorporating mortality events was examined (Supplemental Table 6). The AUC results from this analysis were similar to those obtained using the original Cox proportional hazard model; however, RVFAC and heart rate were more important in the Cox model, whereas breast cancer treatment was more important in the Fine-Gray model. This is most likely due to the causal-specific (etiology of the disease) compared to subdistribution hazard (disease-specific) analysis.

## Discussion

The CHECK HEART-BC study was a multicenter prospective study designed to develop a robust prediction model for cardiotoxicity through systematic evaluation of cardiac function in patients with breast cancer receiving chemotherapy. This study highlights 3 important findings: 1) among the 559 consecutive chemotherapy-naive female patients with breast cancer, cardiotoxicity developed in 46 patients (n = 46 of 559, 8.2%); 2) temporal deterioration in LV and RV systolic function was observed in patients with and without cardiotoxicity; and 3) the risk prediction model (CHECK HEART score) was developed using baseline variables (visit 0) (Central Illustration) and demonstrated sufficient predictive accuracy at 12 months (AUC: 0.82; 95% CI: 0.76-0.89).

**Central Illustration:**
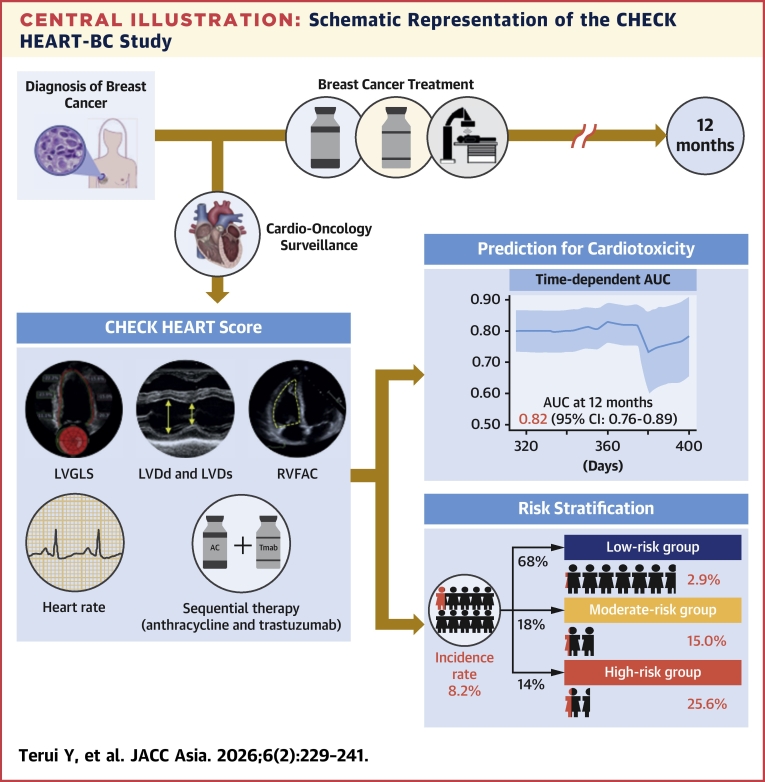
Schematic Representation of the CHECK HEART-BC Study The CHECK HEART score was developed using baseline variables and demonstrated sufficient predictive accuracy at 12 months (time-dependent AUC: 0.82; 95% CI: 0.76-0.89). The formula for the CHECK HEART score was as follows: CHECK HEART score = (0.0269 × [heart rate at baseline, beats/min]) − (0.0384 × [LVDd at baseline, mm]) + (0.209 × [LVDs at baseline, mm]) + (0.199 × [LVGLS at baseline, %]) − (0.0540 × [RVFAC at baseline, %]) + (0.592 × treated with anthracycline and trastuzumab). Notably, approximately 70% of patients were assigned to the low-risk group in which the incidence of cardiotoxicity was only 2.9%. In clinical practice, the follow-up intervals may be extended for patients classified as low risk. Close surveillance is recommended for patients in the moderate- and high-risk groups to enable early detection and improve cardiovascular outcomes. AUC = area under the curve; LVDd = left ventricular diameters in diastole; LVDs = left ventricular diameters in systole; LVGLS = left ventricular global longitudinal strain; RVFAC = right ventricular fractional area change; Tmab = trastuzumab.

## Clinical characteristics of cardiotoxicity

The incidence of cardiotoxicity after chemotherapy in this study (n = 46 of 559, 8.2%) was comparable to rates reported in previous large cohort studies.^21^^,^^22^ Although older age and comorbidities such as hypertension, diabetes mellitus, and dyslipidemia have been identified as risk factors for cardiovascular toxicity,^10^^,^^11^^,^^23^ these factors were similar between patients with and without cardiotoxicity. Ethnicity and background characteristics may have influenced these results, as the study population consisted of relatively young Japanese patients with generally low body mass index. The cardiotoxic effects of anthracycline, trastuzumab, and their sequential use are well established. Consistent with previous studies, the incidence of cardiotoxicity was higher in patients receiving sequential anthracycline and trastuzumab therapy than those not receiving sequential therapy, and this was reflected in the inclusion of sequential therapy within the CHECK HEART score based on literature review. The definition of cardiotoxicity is generally based on LV systolic function; however, significant deterioration in RV systolic function was also observed in this study. Notably, 44 of 46 patients with cardiotoxicity (95.7%) were asymptomatic, whereas only 2 required hospitalization for heart failure. These findings suggested that chemotherapy with or without radiotherapy for breast cancer may lead to stage B heart failure (asymptomatic cardiac diastolic and/or systolic dysfunction), which could progress to symptomatic heart failure (stage C/D). In a previous single-center prospective study, we reported that chemotherapy altered the native T1 value on cardiac magnetic resonance imaging in patients with breast cancer, indicating myocardial damage due to chemotherapy.^24^ Although cardiac magnetic resonance imaging can sensitively detect early-stage heart failure, its clinical application is limited. Therefore, we aimed to develop risk prediction models based on accessible clinical parameters, namely, the CHECK HEART score.

### Predictive value of the CHECK HEART score

Several prediction models have been previously reported; however, they were limited by narrow chemotherapy regimen applicability, small sample sizes, retrospective cohort designs, and insufficient longitudinal cardiac assessment. The present study addressed these limitations by prospectively including patients with breast cancer treated with various regimens and completing both pretreatment and longitudinal cardiac assessment in a large sample size. The CHECK HEART score comprised heart rate, LV end-systolic and end-diastolic diameters, LVGLS, RVFAC, and treatment with anthracycline and trastuzumab. The time-dependent AUC of the risk prediction model suggested sufficient accuracy for clinical practice. The competing risk analysis demonstrated that all-cause mortality did not affect predictive accuracy. Furthermore, the prediction models are feasible because the variables are easily obtainable in clinical settings.

Radiotherapy has been associated with several adverse cardiac effects, including ventricular dysfunction, valvular heart disease, coronary artery disease, and pericardial heart disease.^10^, ^11^, ^12^, ^13^^,^^23^ However, left tangential radiotherapy can reduce cardiac exposure during breast cancer treatment. Anthracyclines and trastuzumab are well-known cardiotoxic agents, and their sequential use has been associated with a high risk of cardiotoxicity in patients with breast cancer.^10^^,^^11^^,^^23^^,^^25^ Consistent with previous studies, sequential anthracycline and trastuzumab therapy synergistically increased cardiotoxicity risk in this study.

Resting heart rate is affected by various factors, including cardiac function, autonomic nervous system, and neurohumoral factors. A high resting heart rate is a known risk biomarker for cardiovascular diseases and cancer. A meta-analysis showed that a resting heart rate of >60 beats/min was a risk factor for poor prognosis, and an increase of 10 to 12 beats/min in the resting heart rate increased the risk in patients with cancer.^26^

In cardio-oncology surveillance, cardiac imaging often focuses on the LV but cardiotoxicity can also lead to RV dysfunction.^27^ Therefore, it is important to evaluate RV function during cardio-oncology surveillance. RVFAC correlated well with RV ejection function and is highly useful in clinical practice.^28^ LVGLS was selected as the parameter for LV systolic function in the risk prediction model based on Cox regression analysis.

Biomarkers, including natriuretic peptides and hs-Tn, were not selected by our analysis as variables. These findings are consistent with a meta-analysis reporting no correlation between natriuretic peptide and LV dysfunction following chemotherapy.^29^ These biomarkers were likely excluded because most patients with cardiotoxicity in this study had asymptomatic LV dysfunction, and no significant changes were detected in echocardiographic parameters related to LV filling pressure, such as E/e′. One hundred ninety-two of 199 patients (96.5%) with elevated serum cardiac troponin levels 3 months after chemotherapy initiation (visit 1) had received anthracycline. Therefore, treatment with anthracycline may be a potential confounder in cardiac troponin elevation, limiting its predictive value for cardiotoxicity development.

### Clinical feasibility of the CHECK HEART score

The incidence of cardiotoxicity varied depending on its validated definition, ranging between 3.7% and 11.4%. Supplemental Figure 3 shows variations in cardiotoxicity definitions across guidelines and studies.^8^^,^^10^^,^^15^^,^^21^ Importantly, the predictive accuracy of the CHECK HEART score remained consistent when applied to the new definitions of cancer therapy–related cardiac dysfunction outlined in the latest European Society of Cardiology guidelines on cardio-oncology.^10^ As shown in the Central Illustration, in clinical practice, the follow-up intervals may be extended for patients classified as low-risk. Approximately 70% (381 of 559) of patients were assigned to the low-risk group. Close surveillance is recommended for patients in the moderate- and high-risk groups (incidence rate of cardiotoxicity: 15.0% [15 of 100] and 25.6% [20 of 78], respectively) to enable early detection and improve cardiovascular outcomes.

### Study limitations

First, although the CHECK HEART score accurately predicted cardiotoxicity during the 12-month follow-up, long-term prognosis remains unknown. Second, the study did not include a control group of patients with breast cancer treated without chemotherapy. Third, although the study population consisted of patients with breast cancer, extrapolation to other cancer types is limited. Additionally, the CHECK HEART score does not account for novel therapeutic agents such as tyrosine kinases inhibitors or immune checkpoint inhibitors. Future studies should target patients receiving these therapies. Fourth, this study lacked external validation, and the generalizability to other racial populations is uncertain. Internal validation demonstrated consistent accuracy, including when cardiotoxicity definitions from various guidelines and studies were applied. A future external validation study is warranted to further assess the utility of the CHECK HEART score.

## Conclusions

The CHECK HEART-BC study, a multicenter prospective study involving comprehensive cardiac assessment in chemotherapy-naïve patients with breast cancer, developed multivariable risk prediction models using the CHECK HEART score. These models accurately predict cardiotoxicity and support effective surveillance in clinical practice.

## Funding Support and Author Disclosures

Supported by Japan Agency for Medical Research and Development (Tokyo, Japan) (18ek0210084h0002 to Dr Sugimura). All other authors have reported that they have no relationships relevant to the contents of this paper to disclose.
