# Supplementary material for: Risk Prediction Models of Cardiotoxicity in Patients With Breast Cancer: Multicenter Prospective CHECK HEART-BC Study
Source: JACC Asia. 2025 Dec 19;6(2):229–41. doi: 10.1016/j.jacasi.2025.08.018 (PMC12904829; doi:10.1016/j.jacasi.2025.08.018)
Supplement: Supplementary Material [file mmc1.docx]

**Supplemental Materials**

Page 3. Supplemental Methods

Page 5. Supplemental Table 1

Univariable Cox Regression Models for the Development of Cardiotoxicity

Page 7. Supplemental Table 2

The Frequency of Variable Selected in 20 Repeated Multivariable Cox Regression Analyses

Page 8. Supplemental Table 3

The secondary outcome (Cardiac adverse events during follow-up period)

Page 9. Supplemental Table 4

Temporal changes in clinical data during follow-up in the patients without cardiotoxicity

Page 11. Supplemental Table 5

Temporal changes in clinical data during follow-up in the patients with cardiotoxicity

Page 13. Supplemental Table 6

Comparison of the original Cox regression and the Fine-Gray competing model

Page 14. Supplemental Figure 1

The Kaplan-Meier curve of cardiotoxicity

Page 16. Supplemental Figure 2

The Kaplan-Meier curve of cardiotoxicity according to chemotherapeutic regimens

Page 20. Supplemental Figure 3

The validation of the CHECK HEART score in case of other definitions of cardiotoxicity

Page 22. References

**Supplemental Methods**

**Echocardiography Image Acquisition and Analysis**

The echocardiography images were acquired by sonographers at the participating institutions. The standard echocardiographic parameters were quantified following the American Society of Echocardiography recommendations.^1, 2^ The left ventricular (LV) ejection fraction (LVEF) was calculated by biplane disk summation, using the apical four- and two-chamber views at the end diastole and end systole, respectively. Endocardial tracing was completed manually, and the LV volume and ejection fraction were calculated automatically. Images for two-dimensional speckle-tracking were acquired in the digital imaging and communications in medicine (DICOM) format. To ensure consistent imaging analysis and measurement quality, the DICOM data were transferred in an anonymized manner to the independent core laboratory at Tohoku University Hospital. LV global longitudinal strain (LVGLS) analysis was performed blindly using TomTec 2D Cardiac Performance Analysis software (TomTec Imaging Systems, Munich, Germany). A total of 2,484 echocardiographic studies were performed on the 559 patients. Of these, 482 were excluded from the LVGLS analysis because of privacy regulations or poor image quality.

**Supplemental Tables**

**Supplemental Table 1. Univariable Cox Regression Models for the Development of Cardiotoxicity**

|  | **Hazard Ratios**  **(95% confidence interval)** | ***P* value** | **Missing Rate (%)** |  |
| --- | --- | --- | --- | --- |
| **Demography** |  |  |  |  |
| Age, yrs. | 1.00 (0.98-1.02) | 0.97 | 0.0 |  |
| Body mass index, kg/m^2^ | 0.97 (0.91-1.05) | 0.46 | 0.2 |  |
| **Cardiovascular Risk Factors** |  |  |  |  |
| Hypertension | 0.71 (0.34-1.46) | 0.35 | 0.0 |  |
| Dyslipidemia | 1.03 (0.54-1.96) | 0.93 | 0.0 |  |
| Diabetes mellitus | 1.03 (0.37-2.87) | 0.96 | 0.0 |  |
| Current or ex-smoker | 0.85 (0.36-2.00) | 0.71 | 0.0 |  |
| History of heart disease | 1.96 (0.77-4.98) | 0.16 | 0.0 |  |
| **Medication** |  |  |  |  |
| Beta-blocker | 2.74 (0.85-8.84) | 0.093 | 0.2 |  |
| RAS inhibitors | 0.93 (0.39-2.19) | 0.87 | 0.2 |  |
| Statins | 1.23 (0.59-3.67) | 0.58 | 0.2 |  |
| **Breast Cancer Characteristics and Treatment** |  |  |  |  |
| Left side | 0.87 (0.49-1.57) | 0.65 | 0.6 |  |
| Early stage | 0.74 (0.39-1.44) | 0.38 | 0.0 |  |
| Triple negative (estrogen, progesterone, her2 receptor) | 0.44 (0.17-1.12) | 0.086 | 0.2 |  |
| Radiotherapy | 1.72 (0.90-3.28) | 0.098 | 1.5 |  |
| Surgery | 1.14 (0.35-3.67) | 0.83 | 0.0 |  |
| Endocrine therapy | 1.25 (0.69-2.25) | 0.47 | 0.4 |  |
| Anthracycline | 1.68 (0.75-3.76) | 0.21 | 0.0 |  |
| Anthracycline dose, mg/m^2^ | 1.00 (1.00-1.01) | 0.42 | 0.0 |  |
| Trastuzumab | 1.51 (0.84-2.73) | 0.17 | 0.0 |  |
| Anthracycline with trastuzumab | 1.64 (0.91-2.94) | 0.097 | 0.0 | |
| Taxan | 1.92 (0.26-13.9) | 0.52 | 0.0 |  |
| **Blood Test (at baseline, Visit 0)** |  |  |  |  |
| Creatinine, mg/dL | 0.62 (0.04-8.83) | 0.73 | 1.5 |  |
| Triglyceride, mg/dL | 1.00 (1.00-1.01) | 0.34 | 4.4 |  |
| LDL cholesterol, mg/dL | 1.00 (0.99-1.01) | 0.33 | 12.4 |  |
| HDL cholesterol, mg/dL | 0.99 (0.97-1.01) | 0.42 | 12.9 |  |
| HbA1c, % | 0.94 (0.63-1.43) | 0.79 | 5.7 |  |
| BNP, pg/mL | 1.01 (1.00-1.02) | 0.26 | 10.7 |  |
| NT-proBNP, pg/mL | 1.00 (0.98-1.03) | 0.75 | 88.2 |  |
| High-sensitive cardiac troponin T, ng/mL | 0.81 (0.17-3.92) | 0.80 | 22.7 |  |
| High-sensitive cardiac troponin I, ng/mL | 0.89 (0.19-4.23) | 0.89 | 82.3 |  |
| **12-Lead ECG (at baseline, Visit 0)** |  |  |  |  |
| Heart rate, bpm | 1.03 (1.00-1.05) | 0.033 | 1.3 |  |
| PR time, msec | 0.99 (0.98-1.01) | 0.36 | 1.8 |  |
| QRS width, msec | 0.99 (0.96-1.02) | 0.50 | 1.5 |  |
| QTc time, msec | 1.00 (0.99-1.02) | 0.49 | 1.5 |  |
| **Echocardiography (at baseline, Visit 0)** |  |  |  |  |
| LVDd, mm | 1.10 (1.02-1.18) | 0.011 | 0.2 |  |
| LVDs, mm | 1.21 (1.11-1.32) | <0.001 | 0.2 |  |
| LAVI, mL/m^2^ | 0.98 (0.95-1.02) | 0.42 | 6.3 |  |
| LVGLS, % | 1.31 (1.10-1.56) | 0.002 | 23.8 |  |
| E/e’ | 0.97 (0.86-1.10) | 0.63 | 0.6 |  |
| RVD, mm | 0.96 (0.89-1.03) | 0.22 | 30.8 |  |
| RVFAC, % | 0.93 (0.87-0.98) | 0.008 | 25.8 |  |
| TAPSE, mm | 0.93 (0.85-1.01) | 0.088 | 11.8 |  |

Hazard ratio was estimated per 0.1 mg/dL increase in creatinine, per 0.001 ng/mL increase in high-sensitive cardiac troponin T and I, respectively.

**Abbreviation;**12-lead ECG, 12-lead electrocardiogram; BNP, B-type natriuretic peptide; CI, confidence interval; LAVI, left atrial volume index; LVDd, left ventricular end-diastolic diameter; LVDs, left ventricular end-systolic diameter; LVGLS, left ventricular global longitudinal strain; NT-proBNP, N-terminal pro-brain natriuretic peptide; RAS inhibitors, renin angiotensin system inhibitors; RVD, right ventricular dimension in diastole; RVFAC, right ventricular fractional area change; TAPSE, tricuspid annular plane systolic excursion.

**Supplemental Table 2. The Frequency of Variable Selected in 20 Repeated Multivariable Cox Regression Analyses**

| **The Frequency of Repetitions** | **Variables** |
| --- | --- |
| 20 of 20 times (100%) | left ventricular end-diastolic diameter |
| 20 of 20 times (100%) | left ventricular end-systolic diameter |
| 20 of 20 times (100%) | left ventricular global longitudinal strain |
| 19 of 20 times (95.0%) | right ventricular fractional area change |
| 16 of 20 times (80.0%) | heart rate |
| 11 of 20 times (55.0%) | tricuspid annular plane systolic excursion |
| 1 of 20 times (5.0%) | LDL cholesterol |

**Supplemental Table 3. The Secondary Outcome (Cardiac Adverse Events during Follow-Up Period)**

| **Patients Number** | **Event** | **Age** | **Chemotherapy** |
| --- | --- | --- | --- |
| 1 | Atrial fibrillation | 74 | AC + Tmab + Taxan |
| 2 | Atrial fibrillation | 75 | AC + Tmab + Taxan |
| 3 | Advanced AV block | 78 | AC + Taxan |
| 4 | Ventricular tachycardia | 61 | AC + Tmab + Taxan |
| 5 | Heart Failure | 56 | AC + Tmab + Taxan |
| 6 | Heart Failure | 58 | Tmab + Taxan |

**Abbreviations;**AC, anthracycline; AV, atrioventricular; Tmab, trastuzumab.

**Supplemental Table 4. Temporal Changes in Clinical Data during Follow-Up in Patients without Cardiotoxicity**

|  | **Visit 0**  **(time point; day 0)**  **N=513** | **Visit 1**  **(time point; day 90±30)**  **N=508** | **Visit 2**  **(time point; day 180±30)**  N=504 | **Visit 3**  **(time point; day 270±30)**  **N=497** | **Visit 4**  **(time point; day 360±30)**  **N=497** |
| --- | --- | --- | --- | --- | --- |
| **Biomarker** |  |  |  |  |  |
| hs-Tn T, ng/mL | 0.005 (0.003, 0.006) | 0.013 (0.007, 0.021) ^**^ | 0.009 (0.006, 0.012) ^**^ | 0.007 (0.005, 0.01) ^**^ | 0.006 (0.005, 0.008) ^**^ |
| hs-Tn I, ng/mL | 0.01 (0.004, 0.01) | 0.01 (0.01, 0.017) ^**^ | 0.01 (0.094, 0.011) | 0.01 (0.008, 0.01) | 0.01 (0.009, 0.01) |
| BNP, pg/mL | 15.2 (8.2, 24.9) | 12.3 (6.2, 24.3) | 12.5 (7.0, 21.8) | 13.2 (7.6, 22.9) | 12.3 (6.8, 21.2) ^*^ |
| NT-proBNP, pg/mL | 73.9 (34.1, 98.9) | 60.0 (28.6, 106) | 57.0 (33.0, 96.5) | 60.6 (36.2, 105) | 57.0 (29.9, 95.8) |
| **12-Lead ECG** |  |  |  |  |  |
| Heart rate, beats per minute | 71.1±11.2 | 77.8±12.1 ^**^ | 75.7±11.8 ^**^ | 70.7±10.3 | 70.8±10.9 |
| PR interval, msec | 156±22 | 155±21 | 158±22 | 160±23 ^*^ | 160±23 ^*^ |
| QRS interval, msec | 92±11 | 91±11 | 91±11 | 92±11 | 92±11 |
| Corrected QT interval, msec | 419±22 | 425±24 ^**^ | 427±23 ^**^ | 425±22 ^**^ | 424±22 ^**^ |
| **Echocardiography** |  |  |  |  |  |
| LVDd, mm | 43.3±4.1 | 44.1±4.1 ^*^ | 44.1±4.2 ^*^ | 44.2±3.9 ^*^ | 44.0±4.2 |
| LVDs, mm | 27.3±3.3 | 28.2±3.4 ^**^ | 28.5±3.3 ^**^ | 28.4±3.3 ^**^ | 28.4±3.6 ^**^ |
| LVEDVi, mL/m^2^ | 82.9±14.8 | 85.8±15.6 ^*^ | 86.0±15.4 ^**^ | 86.2±14.6 ^**^ | 85.4±15.6 |
| LVESVi, mL/m^2^ | 32.2±7.6 | 34.5±8.3 ^**^ | 35.0±7.8 ^**^ | 34.8±8.0 ^**^ | 34.9±8.7 |
| LVEF, % | 67.8±5.4 | 63.9±4.6 ^**^ | 63.2±4.5 ^**^ | 63.2±4.7 ^**^ | 62.6±4.6 ^**^ |
| LAVI, mL/m^2^ | 25.3±8.5 | 27.6±8.8 ^**^ | 27.1±8.5 ^**^ | 27.0±8.9 ^**^ | 26.3±8.5 |
| E/A | 1.11±0.4 | 1.09±0.4 | 1.08±0.4 | 1.07±0.4 | 1.05±0.4 |
| e’, cm/s | 9.6±2.9 | 9.6±2.6 | 9.5±2.6 | 9.3±2.5 | 9.0±2.5 ^**^ |
| E/e’ | 7.8±2.5 | 7.8±2.4 | 7.8±2.5 | 7.8±2.5 | 7.8±2.5 |
| LVGLS, % | -21.4±2.1 | -20.8±2.3 ^**^ | -20.3±2.3 ^**^ | -20.3±2.5 ^**^ | -20.2±2.4 ^**^ |
| RVFAC, % | 46.0±6.5 | 45.4±6.0 | 45.3±6.2 | 45.2±6.4 | 44.7±6.1 ^*^ |
| TAPSE, mm | 21.7±3.9 | 21.7±3.6 | 21.8±3.7 | 21.6±3.7 | 21.1±3.5 ^*^ |

Results are expressed as mean±SD or median (Q1, Q3). The temporal changes in each data from visit 0 to visit 4 were compared by Dunnett’s method using the data at Visit 0 as a reference. Single and double asterisks indicate *P*<0.05 and *P*<0.01, respectively.

**Abbreviations;**12-lead ECG, 12-lead electrocardiogram; BNP, B-type natriuretic peptide; hs-Tn I, High-sensitive Cardiac troponin I; hs-Tn T, High-sensitive Cardiac troponin T; LAVI, left atrial volume index; LVDd, left ventricular diameters in diastole; LVDs, left ventricular diameters in systole; LVEDVi, left ventricular end-diastolic volume index; LVEF, left ventricular ejection fraction; LVESVi, left ventricular end-systolic volume index; LVGLS, left ventricular global longitudinal strain; NT-proBNP, N-terminal pro-brain natriuretic peptide; RVFAC, right ventricular fractional area change; TAPSE, tricuspid annular plane systolic excursion.

**Supplemental Table 5. Temporal Changes in Clinical Data during Follow-Up in Patients with Cardiotoxicity**

|  | **Visit 0**  **(time point; day 0)**  **N=46** | **Visit 1**  **(time point; day 90±30)**  **N=46** | **Visit 2**  **(time point; day 180±30)**  **N=46** | **Visit 3**  **(time point; day 270±30)**  **N=46** | **Visit 4**  **(time point; day 360±30)**  **N=46** |
| --- | --- | --- | --- | --- | --- |
| **Biomarker** |  |  |  |  |  |
| hs-Tn T, ng/mL | 0.004 (0.003, 0.006) | 0.02 (0.01, 0.02) ^**^ | 0.01 (0.006, 0.02) ^**^ | 0.008 (0.005, 0.01) ^**^ | 0.006 (0.004, 0.009) ^**^ |
| hs-Tn I, ng/mL | 0.01 (0.006,0.01) | 0.01 (0.01, 0.02) | 0.01 (0.008, 0.02) | 0.01 (0.007, 0.01) | 0.01 (0.006, 0.01) |
| BNP, pg/mL | 14.8 (8.7, 27.9) | 12.3 (7.5, 29.2) | 12.2 (7.4, 23.2) | 14.8 (10.1, 27.1) | 12.3 (9.5, 27.0) |
| NT-proBNP, pg/mL | n/a | 145 (141, 148) | 221 (185, 2109) | 123 (86, 159) | 207 (113, 292) |
| **12-Lead ECG** |  |  |  |  |  |
| Heart rate, beats per minute | 74.4±10.5 | 86.0±13.0 ^**^ | 79.8±11.7 | 74.6±43.4 | 73.0±11.1 |
| PR interval, msec | 153±22 | 151±21 | 155±20 | 156±24 | 158±22 |
| QRS interval, msec | 91±13 | 90±13 | 89±11 | 89±7 | 91±13 |
| Corrected QT interval, msec | 422±27 | 430±30 | 428±30 | 428±23 | 429±26 |
| **Echocardiography** |  |  |  |  |  |
| LVDd, mm | 45.0±3.9 | 46.8±4.4 | 46.7±4.8 | 46.3±4.7 | 47.4±4.5 ^*^ |
| LVDs, mm | 29.6±3.3 | 31.4±4.1 | 32.9±5.0 ^**^ | 32.8±4.8 ^**^ | 33.8±5.6 ^**^ |
| LVEDVi, mL/m^2^ | 90.0±15.4 | 97.8±19.2 | 97.3±23.0 | 96.0±21.8 | 100±20.4 |
| LVESVi, mL/m^2^ | 38.3±8.9 | 43.5±12.7 | 48.3±18.6 ^*^ | 47.9±16.9 ^**^ | 51.1±19.4 ^**^ |
| LVEF, % | 65.9±7.0 | 57.2±7.1 ^**^ | 53.4±9.1 ^**^ | 52.2±7.2 ^**^ | 50.0±10.3 ^**^ |
| LAVI, mL/m^2^ | 24.3±5.7 | 26.7±7.4 | 26.9±7.5 | 26.1±7.6 | 29.7±10.3 ^*^ |
| E/A | 1.04±0.4 | 1.08±0.4 | 1.04±0.3 | 1.00±0.3 | 1.02±0.3 |
| e’, cm/s | 9.3±2.6 | 9.3±2.6 | 9.0±2.2 | 8.9±2.2 | 8.5±2.3 |
| E/e’ | 7.6±2.5 | 7.7±2.0 | 7.7±2.1 | 7.5±2.4 | 8.4±3.3 |
| LVGLS, % | -20.5±1.9 | -19.2±3.1 | -17.9±2.8 ^*^ | -17.9±2.1 ^**^ | -18.5±3.0 ^**^ |
| RVFAC, % | 43.2±5.9 | 41.8±7.1 | 42.4±6.9 | 41.5±7.0 | 39.7±7.2 ^*^ |
| TAPSE, mm | 20.7±3.4 | 20.9±3.3 | 19.9±3.9 | 20.3±4.3 | 19.8±4.9 |

Results are expressed as mean±SD or median (Q1, Q3). The temporal changes in each data from visit 0 to visit 4 were compared by Steel’s method using the data at Visit 0 as a reference. Single and double asterisks indicate *P*<0.05 and *P*<0.01, respectively.

**Abbreviations;**12-lead ECG, 12-lead electrocardiogram; BNP, B-type natriuretic peptide; hs-Tn I, High-sensitive Cardiac troponin I; hs-Tn T, High-sensitive Cardiac troponin T; LAVI, left atrial volume index; LVDd, left ventricular diameters in diastole; LVDs, left ventricular diameters in systole; LVEDVi, left ventricular end-diastolic volume index; LVEF, left ventricular ejection fraction; LVESVi, left ventricular end-systolic volume index; LVGLS, left ventricular global longitudinal strain; NT-proBNP, N-terminal pro-brain natriuretic peptide; RVFAC, right ventricular fractional area change; TAPSE, tricuspid annular plane systolic excursion.

**Supplemental Table 6. Comparison of the original Cox regression and the Fine-Gray competing model**

|  | **The Original Cox Model** | | **The Fine-Gray Competing Risk Model** | |
| --- | --- | --- | --- | --- |
|  | **Hazard Ratios**  **(95% confidence interval)** | **P value** | **Hazard Ratios**  **(95% confidence interval)** | **P value** |
| **Echocardiography (at baseline, Visit 0)** |  |  |  |  |
| LVDd, mm | 0.96 (0.86-1.08) | 0.52 | 0.96 (0.86-1.07) | 0.46 |
| LVDs, mm | 1.23 (1.07-1.41) | 0.005 | 1.23 (1.11-1.37) | <0.001 |
| LV GLS, % | 1.22 (1.04-1.43) | 0.020 | 1.23 (1.09-1.38) | 0.001 |
| RVFAC, % | 0.95 (0.90-1.00) | 0.059 | 0.95 (0.85-1.05) | 0.33 |
| **12-Lead ECG (at baseline, Visit 0)** |  |  |  |  |
| Heart rate, bpm | 1.03 (1.00-1.05) | 0.034 | 1.03 (0.92-1.14) | 0.62 |
| **Breast Cancer Treatment** |  |  |  |  |
| Anthracycline with trastuzumab | 1.81 (0.99-3.29) | 0.060 | 1.82 (1.59-2.07) | <0.001 |

**Abbreviations;**12-lead ECG, 12-lead electrocardiogram; LVDd, left ventricular end-diastolic diameter; LVDs, left ventricular end-systolic diameter; LV GLS, left ventricular global longitudinal strain; RVFAC, right ventricular fraction area change.

**Supplemental Figures and Figure Legends**

**Supplemental Figure 1. The Cumulative Incidence Curves of Cardiotoxicity**

**Panel A.**

**Panel B.**

Panel A. Cardiotoxicity defined as a reduction of >10% point in the left ventricular ejection fraction from baseline to a value <53% measured by echocardiography developed in 46 (8.2%) during follow-up period (median follow-up of 366 days, Q1-Q3: 365 to 367). Panel B shows the original cumulative incidence curve using the Fine-Gray model.

**Supplemental Figure 2. The Kaplan-Meier Curve of Cardiotoxicity According to Chemotherapeutic Regimens**

**Panel A.**

**Panel B**

**Panel C.**

**Panel D.**

Each panel shows the Kaplan-Meier curve of cardiotoxicity according to chemotherapeutic regimens. Panel D shows a tendency to more incidence of cardiotoxicity in the patients treated with anthracycline and trastuzumab.

**Supplemental Figure 3. The Validation of the CHECK HEART Score in Case of Other Definitions of Cardiotoxicity**

The accuracy of the CHECH HEART score was demonstrated by evaluating different definitions of cardiotoxicity. The blue line (reduction of >10% in LVEF from baseline to an LVEF value of <53%) shows the time-dependent area under the receiver operating characteristic (time-dependent AUC) curves based on the primary outcome. The time-dependent AUC was consistent with the curves based on the new definition of cancer therapy-related cardiac dysfunction in the latest European Society of Cardiology guidelines (green line: reduction of >10% in LVEF from baseline to an LVEF value of <50%). There was no significant difference between the blue line and any of the other lines.

**References**

1. Mitchell C, Rahko PS, Blauwet LA, et al. Guidelines for performing a comprehensive transthoracic echocardiographic examination in adults: Recommendations from the American Society of Echocardiography. J Am Soc Echocardiogr 2019;32:1-64.

2. Nagueh SF, Smiseth OA, Appleton CP, et al. Recommendations for the evaluation of left ventricular diastolic function by echocardiography: An Update from the American Society of Echocardiography and the European Association of Cardiovascular Imaging. Eur Heart J Cardiovasc Imaging 2016;17:1321-1360.
